# Diagnosis and management of acute abnormal uterine bleeding during menacme

**DOI:** 10.1016/j.clinsp.2025.100608

**Published:** 2025-03-09

**Authors:** Gabriela Pravatta-Rezende, Cristina Laguna Benetti-Pinto, Daniela Angerame Yela Gomes, Ana Carolina Japur de Sá Rosa e Silva, José Maria Soares

**Affiliations:** a Department of Obstetrics and Gynecology, Faculdade de Ciências Médicas, Universidade Estadual de Campinas (UNICAMP), Campinas, SP, Brazil; b Universidade Estadual de Campinas (UNICAMP), Campinas, SP, Brazil; c Faculdade de Medicina de Ribeirão Preto (FMRP), Universidade de São Paulo (USP), Ribeirão Preto, SP, Brazil; d Universidade de São Paulo (USP), São Paulo, SP, Brazil

**Keywords:** Abnormal uterine bleeding, Menorrhagia, Acute bgynecological conditions, Menstrual disorders

## Abstract

**Objective:**

To provide a protocol for the diagnosis and management of Acute Abnormal Uterine Bleeding (AUB) during menacme, addressing common causes, clinical evaluation, and treatment options.

**Methods:**

A review of current evidence and guidelines was performed to create a structured approach for healthcare professionals.

**Results:**

Acute AUB, defined as excessive uterine bleeding unrelated to pregnancy, requires immediate intervention. Causes vary by age and include coagulopathies, anovulation, and structural anomalies. Clinical stability, lab workups, and imaging are pivotal in guiding management. Initial treatment focuses on hemodynamic stabilization followed by medical therapies such as antifibrinolytics, hormonal agents, or surgical intervention when necessary.

**Conclusion:**

Early intervention in AUB ensures optimal outcomes, reduces complications, and allows transition to maintenance therapy to prevent recurrence.

## Highlights

• Abnormal Uterine Bleeding (AUB) is the leading cause of seeking gynecological care, with data showing that 3 in 10 Brazilian women have this condition, with intermenstrual bleeding standing out.
• According to the International Federation of Gynecology and Obstetrics (FIGO), cycles lasting less than 24 days or more than 38 days, with menstrual flow lasting eight days or more and blood loss reported by the woman as intense, are considered abnormal.
• The main causes of AUB are summarized in the mnemonic PALM-COEIN, dividing them into structural and non-structural causes.
• Diagnosing the etiology of AUB is essential to establish specific treatments, directed at the cause, which can be clinical or surgical, aiming not only to control the amount of blood loss, but also to improve the quality of life of women with AUB.
• In general, the first line of treatment for AUB is medication, with surgical treatment being intended for failure or contraindication to clinical treatment or in cases of significant hemodynamic instability.

## Introduction

Acute Abnormal Uterine Bleeding (AUB-a) is defined as excessive blood flow originating from the uterus, unrelated to pregnancy, requiring immediate intervention to reduce blood loss and prevent clinical and hemodynamic instability.^1^ It may present as an isolated episode or as an acute manifestation of a chronic condition, often requiring hospitalization and increasing healthcare costs.^2^

AUB can be caused by polyps, adenomyosis, leiomyomas, endometrial hyperplasia or malignancy, coagulopathy, ovulatory dysfunction, endometrial causes, iatrogenic factors, and a final group comprising unclassified causes, grouped under the acronym PALM-COEIN.^3^ Regarding acute bleeding, the disorders most frequently associated vary by age (Table 1). Ovulatory disorders predominantly occur in the early years following menarche, mainly due to the immaturity of the hypothalamic-pituitary-ovarian axis, and during the menopausal transition phase. However, other conditions can also present with anovulatory cycles, such as polycystic ovary syndrome, thyroid disorders, and hyperprolactinemia, which may manifest as acute bleeding symptoms.^4^, ^5^, ^6^, ^7^

**Table 1:** Most prevalent causes of acute abnormal uterine bleeding stratified by age.

**Age**	Most Prevalent Causes
**Adolescents**	Coagulopathies
	Anovulation (immaturity of the hypothalamic-pituitary-ovarian axis)
**Adults (< 40 years)**	Structural causes (polyps and fibroids)
	Ovulatory causes (polycystic ovary syndrome, hyperprolactinemia)
**Perimenopause (> 40 years)**	Structural causes (polyps, fibroids, endometrial hyperplasia, and malignancy)
	Anovulation (ovarian insufficiency)
**Postmenopause**	Endometrial atrophy
	Structural causes (polyps, endometrial hyperplasia, malignancy)

Source: Translated and adapted from Kelly B, Buttigieg E. Evaluation and Management of Heavy Vaginal Bleeding (Noncancerous). Obstet Gynecol Clin North Am. 2022;49(3):591–606.(6).

Coagulopathies are another cause of AUB-a, with evidence showing their presence in 10% to 34% of women with increased menstrual volume since menarche or other types of bleeding, such as epistaxis and gingival bleeding. Von Willebrand disease is the most common coagulopathy.^7^, ^8^, ^9^ Given the relative scarcity of evidence in the treatment of Acute Abnormal Uterine Bleeding (AUB-a), this protocol aims to propose a management and care pathway for emergency or urgent cases. This approach applies provided that gestational causes of bleeding have been reliably excluded.

## Clinical evaluation and diagnosis

At the initial evaluation of women with Acute Abnormal Uterine Bleeding (AUB-a), upon admission, the approach focuses on assessing vital signs and hemodynamic stability (particularly pulse, blood pressure, and mucosal color), in addition to ruling out pregnancy. Hemodynamic stability can be evaluated through the palpation of peripheral pulses, blood pressure, heart rate, level of consciousness, and urinary output, as described in Table 2.

**Table 2:** Classes of hypovolemic shock.^6^^-^^8^

**Class**	**Class 1**	**Class 2**	**Class 3**	**Class 4**
Heart Rate (bpm)	< 100	> 100	> 120	> 140
Blood Pressure (mmHg)	≥ 120 × 80	≥ 120 × 80	< 120 × 80	< 120 × 80
Respiratory Rate (breaths/min)	14‒20	20‒30	30‒35	>35
Consciousness State	Mildly agitated	Moderately agitated	Agitated, confused	Lethargic
Urine Output (mL/h)	> 30	20‒30	5‒15	< 5
Estimated Blood Loss	< 15%; 750 mL	15%‒30%; 750‒1500 mL	30%‒40%; 1500‒2000 mL	> 40%; > 2000 mL

In general, women with Acute Abnormal Uterine Bleeding (AUB-a) seek medical care when blood loss is significant enough to cause symptoms related to bleeding. Approximately 35% of these patients present with anemia at the time of consultation, with hemoglobin levels below 10 g/dL observed in 13.7% of cases. Based on clinical findings, hematimetric evaluation (hemoglobin and hematocrit) is indicated. This assessment will determine the need for intravenous access for volume replacement (crystalloids, preferably Ringer's lactate) and, if necessary, transfusion of blood products.^10^

Simultaneously, a thorough medical history should be taken, detailing the bleeding onset, duration, characteristics, and volume. Personal history should address prior episodes of AUB, comorbidities, previous surgeries, habits, gynecological and obstetric history, with particular attention to menstrual cycle regularity, contraceptive use, and medications, especially antipsychotics, antidepressants, and antiepileptics that may interfere with hormone production and ovulation, as well as anticoagulants. Clinical and gynecological examinations should also be performed.^11^

Regarding gynecological evaluation, a speculum examination is essential to assess and quantify bleeding, as well as to identify cervical lesions, polyps, or fibroids protruding through the cervicals. The bimanual examination should evaluate uterine size, adnexal palpation, and other pelvic abnormalities (Table 3).

**Table 3:** Information obtained from general and gynecological examinations that may aid in the diagnosis of AUB-a in non-pregnant women.

**Examination**	**Findings**
General Examination	Temperature, blood pressure, pulse, weight, body mass index, assessment of mucosa (color, hydration, bleeding).
Skin	Petechiae, ecchymosis, hirsutism, acne, acanthosis nigricans.
Neck	Thyroid examination.
Genital Organs	(Inspection or, when possible, speculum and bimanual examination):
	- Amount of bleeding.
	- Trauma or lesions with vulvar, vaginal, urethral, or anal bleeding.
	- Increased uterine size or irregularity, adnexal volume.

To determine the likely etiology of AUB-a, the classification system suggested by PALM-COEIN should be adopted. Clinical reasoning may indicate the need for imaging or additional laboratory tests. Transvaginal ultrasound can aid in diagnosing structural abnormalities of the uterus. Endometrial biopsy (methods: pipelle, Novak, hysteroscopy, or uterine curettage) is recommended for women at higher risk of hyperplasia and endometrial malignancy, especially those over 40-years old, with a history of prolonged anovulation, diabetes, obesity, family history of endometrial cancer, prolonged exposure to unopposed estrogen, or tamoxifen use.^12^, ^13^, ^14^

Early treatment initiation for AUB-a is recommended, with the primary goal being the control of bleeding to prevent more severe consequences.

## Management and treatment

The primary goals guiding the treatment of Acute Abnormal Uterine Bleeding (AUB-a) are to control the current bleeding, stabilize the patient, and reduce the risk of excessive blood loss in subsequent cycles. After achieving hemodynamic stabilization, clinical treatment options are divided into hormonal and non-hormonal medications. In some cases, surgical intervention may be necessary, including procedures such as endometrial tamponade, dilation and curettage, hysteroscopy, endometrial ablation, uterine artery embolization, or hysterectomy.

Hysterectomy is considered a last-resort therapeutic option and should take into account the woman's reproductive desires and whether her family planning is complete.

### Non-hormonal treatment

#### Antifibrinolytics

In cases of abnormal uterine bleeding, antifibrinolytics are often considered, with tranexamic acid being a first-line treatment, reducing reported menstrual blood loss by 34% to 54%. It can be administered orally or parenterally, either alone or in combination with hormonal therapies.^15^, ^16^, ^17^ It is also effective in reducing blood loss secondary to coagulopathies, with contraindications limited to acute thromboembolic vascular disease and a history of hypersensitivity to its components.^18^ Commonly reported side effects include headache, lower back pain, abdominal pain, and fatigue.^19^

The recommended oral dose of tranexamic acid is 1.5*g* to 4*g* per day, with an initial suggested dose of 500 mg every 8 h for 3 to 5 days, which can be increased to a maximum of 4*g*. For intravenous use, in cases requiring hospitalization, a dose of 10 mg/kg every 8 h for 3 to 5 days is recommended.^20^

Although less studied, another antifibrinolytic option is aminocaproic acid, preferably administered intravenously at a loading dose of 4*g* to 5*g* , followed by a maintenance dose of 1*g*/hour for a maximum of 24 h, reserved for in-hospital treatment.^21^

Intravenous antifibrinolytic therapy is recommended in AUB-a cases, particularly in the presence of hemodynamic instability.

#### Nonsteroidal anti-inflammatory drugs (NSAIDs)

Through the inhibition of prostaglandins, NSAIDs can reduce uterine bleeding and relieve pelvic discomfort. They can be used in combination with antifibrinolytics and hormonal treatments, but are less effective when used alone. The most commonly used drugs include mefenamic acid, naproxen, ibuprofen, flurbiprofen, and diclofenac. Few studies have compared NSAIDs directly, with no evidence of superiority among them. The most frequent side effects are gastrointestinal, though rarely severe.^22^

Some NSAIDs with proven efficacy for managing acute and chronic bleeding are described in Table 2.^3^

### Hormonal treatment

Hormonal medications are considered first-line therapy for women with Acute Abnormal Uterine Bleeding (AUB-a) and include Combined Hormonal Contraceptives (CHCs) and oral progestogens.

Although not currently available in Brazil, high-dose intravenous estrogen (25 mg every 4 to 6 h for 24 h) should be noted as it rapidly induces endometrial growth, stimulates uterine artery contraction, promotes platelet aggregation, and coagulation. The literature demonstrates bleeding control in 72% of cases.^23^ Ulipristal acetate should also be mentioned as it has been shown to rapidly induce amenorrhea in women with uterine fibroids, potentially serving as a useful treatment for acute bleeding emergencies related to fibroids.^24^

#### Combined hormonal contraceptives (CHCs)

The use of CHCs is recommended for the treatment of AUB-a in the absence of contraindications to estrogen. They can also be used as maintenance therapy after stabilization of the acute episode. The most studied and therapeutically effective formulations are monophasic, containing ethinylestradiol combined with progestogens.^18^ Due to the lack of robust scientific evidence comparing available treatments for AUB-a, Table 4 has been prepared based on treatments cited in references 11, 17, 22, and 24.

**Table 4:** Formulations and dosages of the main NSAIDs used in the management of AUB.

**Formulations**	**Doses**
Mefenamic Acid	500 mg every 12-hours or every 8-hours, for 3‒5 days
	100 mg/kg of body weight, every 8-hours, for 3 to 5 days, if hospitalization is required
Naproxen	500 mg every 12-hours, for 3‒5 days
	500 mg in the morning and 250 mg at night for 2-days, followed by 250 mg every 12-hours for 7-days
Ibuprofen	800 mg every 8-hours, for 5-days
Diclofenac	500 mg every 8-hours, for 5-days (or up to 150 mg/day)

Source: Translated and adapted from Munro MG, Mainor N, Basu R, Brisinger M, Barreda L. Oral medroxyprogesterone acetate and combination oral contraceptives for acute uterine bleeding: a randomized controlled trial. Obstet Gynecol 2006;108(4):924–9.(3).

It is worth noting that high-estrogen-dose contraceptives, such as ethinylestradiol 35 mg, do not demonstrate superiority in controlling acute bleeding and are associated with a higher risk of side effects.^25^ Additionally, there is insufficient evidence to recommend contraceptives with natural estrogens, such as estradiol valerate or estradiol, for AUB-a.

Although not classified as a contraceptive, another possible medication is the combination of ethinylestradiol 0.05 mg and cyproterone acetate 10 mg, administered at 1 tablet 3 times daily with gradual reduction based on symptom control, while adhering to contraindications.

#### Isolated progestogens

The oral use of medroxyprogesterone acetate (currently with limited availability in Brazil) and norethisterone is also validated for the treatment of acute abnormal uterine bleeding (AUB-a) due to their mechanism of inhibiting endometrial proliferation. In cases where estrogens are contraindicated, isolated progestogens are particularly indicated.

Levonorgestrel intrauterine systems, etonogestrel subdermal implants, and depot medroxyprogesterone acetate are formulations containing isolated progestogens that can be used as maintenance treatments for abnormal bleeding, but they are not suitable for managing acute episodes.^1^^,^^3^^,^^25^

The formulations and doses are summarized in Table 5, Table 6, highlighting that it is possible to establish either isolated non-hormonal or hormonal treatments, as well as to combine both therapeutic modalities when appropriate.^26^, ^27^, ^28^, ^29^

**Table 5:** Formulations and dosages of the main hormonal treatments used in the clinical management of AUB.

**Suggested Formulation**	**Loading Dose**	**Maintenance Dose**
Combined Oral Contraceptive (30 µg EE + progestogen - norethisterone, levonorgestrel, gestodene)	One tablet every 6‒8 h until bleeding stops (maintain for at least two days)	One tablet daily for three to six weeks
	OR	
	One tablet every 8-hours for two to seven days, followed by one tablet every 12-hours for two to seven days, then one tablet daily for at least four weeks	

Precautions:.

Do not prescribe to women with contraindications to estrogen.

Main side effect: nausea (consider antiemetics).

Reassess response within 48 to 72 h.

**Table 6:** Formulations and dosages of the main progestogens used alone in the management of AUB.

**Formulations**	**Loading Dose**	**Maintenance Dose**
Medroxyprogesterone Acetate	60 to 120 mg/day, orally, until bleeding stops (for at least 2 days)	Followed by 20 to 40 mg/day, for 3 to 6 weeks
Medroxyprogesterone Acetate	10 mg every 4 h, orally, until bleeding stops	Followed by 10 mg orally every 6 h for 4 days, then every 8 h for 3 days, every 12 h for 2 days, daily for 3 to 6 weeks
Norethisterone	5 to 15 mg/day, orally, until bleeding stops (for at least 2 days)	Followed by 5 to 10 mg/day, for 3 to 6 weeks
Norethisterone	5 to 10 mg every 4 h, orally, until bleeding stops	Followed by 5 to 10 mg every 6 h for 4 days, then every 8 h for 3 days, every 12 h for 2 days, daily for 3 to 6 weeks
Megestrol Acetate	80 to 160 mg/day, orally, until bleeding stops (for at least 2 days)	Followed by 40 to 80 mg/day, for 3 to 6 weeks

Source: Adapted from Munro MG; Southern California Permanente Medical Group's Abnormal Uterine Bleeding Working Group. Acute uterine bleeding unrelated to pregnancy: a Southern California Permanente Medical Group practice guideline. Perm J. 2013;17(3):43–56; El-Hemaidi I, Gharaibeh A, Shehata H. Menorrhagia and bleeding disorders. Current Opinion in Obstetrics and Gynecology 2007;19(6):513–20.^20^^,^^24^

#### Coagulopathies and patients on anticoagulants

These situations pose significant challenges in the management of Acute Abnormal Uterine Bleeding (AUB-a), as they often contraindicate the use of estrogens. A multidisciplinary evaluation is strongly recommended. Desmopressin, administered intranasally, subcutaneously, or intravenously, can be used in cases of AUB-a secondary to von Willebrand disease.^28^^,^^29^

### Procedures and surgical treatments

Surgical treatments are considered second-line options in AUB-a and are reserved for cases refractory to clinical treatment or AUB secondary to structural causes, particularly submucosal leiomyomas and endometrial polyps.

#### Intrauterine tamponade

The use of intrauterine balloons is well-known in obstetrics for postpartum hemorrhage, particularly the Bakri balloon, which is employed in cases of uterine atony. Literature also reports the use of a Foley catheter inflated with distilled water or saline for controlling acute, non-gestational bleeding, especially in adolescents with coagulopathies refractory to clinical treatment. This technique shows promise as a low-cost, low-risk therapeutic option. The recommendation is for the balloon to remain in the uterine cavity for 2 to 48 h, with continuous clinical reassessment. A size 26 Foley catheter with 20 to 30 mL of saline is suggested. However, robust evidence is lacking to recommend this as a routine approach.^30^, ^31^, ^32^

#### Dilation and curettage/hysteroscopy

Dilation and curettage are reserved options due to the risk of adhesion formation. However, in some settings, they may be a necessary consideration. It is important to emphasize that curettage may not address structural lesions causing the bleeding, as it is performed “blindly”. When possible, hysteroscopy should be prioritized for visualizing the uterine cavity and targeting the treatment of focal lesions such as polyps and submucosal leiomyomas. Furthermore, using a resectoscope, endometrial ablation becomes a viable option. However, data are scarce regarding its use in acute cases, with limited evidence on the best timing for its application, as well as challenges in visualizing lesions during active bleeding.^33^, ^34^, ^35^, ^36^ Both curettage and hysteroscopy are adequate for histopathological evaluation of the endometrium. Options such as intrauterine balloons and radiofrequency ablation are also available, though not always accessible.^37^

#### Uterine artery embolization

This technique is used in certain cases of obstetric hemorrhage but with limited data on its application in AUB-a. It may be considered when clinical treatment fails in women with contraindications to surgical approaches or in cases where fertility preservation is desired. Reports suggest that embolization might be the most appropriate therapeutic option in the presence of arteriovenous malformations.^38^^,^^39^

#### Hysterectomy

**Fig. 1:**
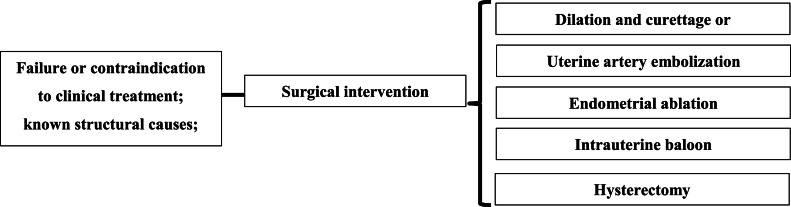
Summary of the main surgical interventions in AUB. Source: translated, adapted, and modified from Munro MG; Southern California Permanente Medical Group's Abnormal Uterine Bleeding Working Group. Acute uterine bleeding unrelated to pregnancy: a Southern California Permanente Medical Group practice guideline. Perm J. 2013;17(3):43–56.^20^

Hysterectomy is considered the last option for women with AUB-a and is generally limited to cases of treatment failure or contraindications to medical therapy, or when the severity of bleeding warrants this approach. It may also be performed in response to underlying medical conditions. Hysterectomy can be carried out via laparotomy, laparoscopy, or vaginally.^40^^,^^41^ This option must be carefully considered, particularly in women who have not completed childbearing (Fig. 1).^11^

## Conclusion

In cases of Acute Abnormal Uterine Bleeding (AUB-a), the severity of the bleeding dictates the urgency and focus of care, which may necessitate transfusions, hospitalization, or the possibility of outpatient treatment. Whenever feasible, the management of AUB-a should initially prioritize medical treatment. Treatment choices are guided by the patient's history, clinical examination, medical background, and contraindications, as well as considering reproductive desires and the severity of the bleeding, which determines hemodynamic status.

Once the acute bleeding episode is controlled, transitioning to maintenance therapy and referring the patient for outpatient evaluation should be considered. If prompt outpatient follow-up is not feasible, continuing medication for three cycles is advisable to prevent recurrence. Surgical treatment is reserved for cases of clinical instability, lack of response to medical therapy, or the presence of contraindications to medical treatment.

## Declaration of competing interest

The authors declare no conflicts of interest.
